# Editorial: Strategies aimed at improving the effectiveness of immunotherapies in pediatric brain cancer

**DOI:** 10.3389/fimmu.2025.1771946

**Published:** 2026-01-19

**Authors:** Samuele Tardito, Dalia Haydar, Yi Cao

**Affiliations:** 1 Brain Tumor Institute, Center for Cancer and Immunology Research, Children’s National Hospital, Washington, WA, United States; 2 Center for Cancer and Immunology Research, Children’s National Hospital, Washington, WA, United States; 3 Key Laboratory of Environment-Friendly Chemistry and Application of Ministry of Education, Lab of Biochemistry, College of Chemistry, Xiangtan University, Xiangtan, China

**Keywords:** CAR (chimeric antigen receptor) T cells, immunotherapy, iPSC (induced pluripotent stem cell), pediatric brain cancer, tumor microenvironment

Pediatric brain tumors are biologically distinct from adult cancers, often arising during development and presenting diverse histological and molecular profiles. Their treatment is complicated by the blood-brain barrier, the brain’s delicate structure, and limited regenerative capacity. While immunotherapy has revolutionized adult oncology, its use in pediatric brain tumors is still emerging due to an immunosuppressive tumor microenvironment and limited immune profiling. This Research Topic highlights recent advances in understanding the pediatric brain TME and explores innovative models and immunotherapeutic strategies to improve outcomes for young patients.

## 1 Thematic summary of contributions

### 1.1 Tumor microenvironment and immune profiling in medulloblastoma

Understanding the immune landscape of pediatric brain tumors is essential for developing effective immunotherapies. Chen et al. profiled medulloblastoma (MB) subgroups WNT, SHH, Group 3, and Group 4 revealing distinct immune cell infiltration patterns. Group 3 tumors showed elevated CD8+ T cells, Tregs, and endothelial cells. Three hub genes (GAB1, CXCR4, ABL1) were identified as potential biomarkers linked to immune checkpoint responsiveness and drug sensitivity, supporting subgroup-specific immunotherapy strategies. Bhargav et al. highlighted the importance of modeling the pediatric brain tumor microenvironment to improve translational relevance. Their review outlines advanced platforms organoids, organotypic brain slices, and 3D bioengineered systems that better replicate immune-tumor interactions. These models are essential for evaluating immunotherapies and overcoming barriers in MB and glioma.

### 1.2 CAR-T cell therapy: overcoming trafficking and persistence barriers

CAR-T cell therapy holds promise for pediatric brain malignancies but faces significant challenges. Yaacoub et al. reviewed two major obstacles: poor trafficking across the blood-brain barrier (BBB) and limited persistence in the immunosuppressive tumor microenvironment. They discussed locoregional delivery methods, focused ultrasound, and biomaterials to enhance trafficking, alongside CAR engineering strategies co-stimulatory domains, cytokine support, and checkpoint inhibition to improve persistence and efficacy.

### 1.3 T cell differentiation from induced pluripotent stem cells

Generating functional T cells from induced pluripotent stem cells (iPSCs) offers a scalable source for adoptive cell therapies. Ishiguro et al. compared 2D feeder-free and 3D organoid cultures for differentiating T cells from T-iPSCs. While 2D systems produced CD8+ T cells, only 3D organoids generated mature CD4+ T cells, recapitulating thymic development. Single-cell RNA sequencing and CITE-seq identified key transcriptional regulators (ZBTB7B, BCL11B, GATA3, RUNX3) involved in lineage commitment, advancing scalable helper T cell production.

### 1.4 Immunotherapy modalities in glioma

Gliomas, particularly glioblastoma (GBM), remain challenging to treat due to their immunosuppressive microenvironment and heterogeneity. Yasinjan et al. reviewed immunotherapy strategies including checkpoint inhibitors, CAR-T cells, vaccines, and oncolytic viruses. While ICIs have shown limited efficacy, neoadjuvant PD-1 blockade and DC vaccines (e.g., DCVax-L) show promise. CAR-T cells targeting EGFRvIII, HER2, and IL13Rα2 are under investigation, and the conditional approval of oncolytic virus G47Δ in Japan marks a clinical milestone.

### 1.5 Comparative immunogenomics of pediatric vs. adult high-grade gliomas

Aggarwal et al. compared pediatric (pHGG) and adult high-grade gliomas (aHGG), highlighting distinct molecular and immunological profiles. Pediatric gliomas often harbor histone mutations (e.g., H3K27M), while adult gliomas are characterized by IDH mutations and EGFR amplification. Both exhibit immunosuppressive TMEs, but pediatric tumors tend to be “immune cold.” Shared targets like GD2 and EGFRvIII offer therapeutic overlap, though age-specific strategies remain essential.

### 1.6 Medulloblastoma biology and immunotherapy targets

Poggi et al. reviewed MB biology and immunotherapy targets, including tumor-associated antigens and neoantigens. They discussed the role of innate immune cells (e.g., NK cells) and unconventional T cells in MB immunosurveillance, and the potential of CAR-T cells and monoclonal antibodies. Advances in single-cell transcriptomics and preclinical models are paving the way for precision immunotherapy tailored to MB subgroups.

## 2 Cross-cutting insights and emerging trends

The articles in this Research Topic collectively underscore several converging challenges and innovations in pediatric brain cancer immunotherapy. A recurring theme is the complexity of the tumor microenvironment (TME), which remains a major barrier to effective immune engagement. Both Chen et al. and Bhargav et al. emphasize the need for subgroup-specific immune profiling and physiomimetic models to better understand and overcome immune suppression in medulloblastoma and glioma.

CAR-T cell therapy, as discussed by Yaacoub et al., faces dual limitations in trafficking and persistence. These challenges are echoed in the modeling strategies reviewed by Bhargav et al., who advocate for platforms that can simulate immune cell infiltration and exhaustion dynamics. The integration of delivery innovations such as focused ultrasound and biomaterials with advanced CAR engineering represents a promising frontier.

The potential of induced pluripotent stem cells (iPSCs) to generate functional T cells, as shown by Ishiguro et al., opens new avenues for scalable and customizable cell therapies. This approach complements efforts to personalize immunotherapy based on molecular subgrouping, as highlighted by Chen et al., Aggarwal et al., and Poggi et al.

Emerging trends include the use of neoantigen discovery tools, combination therapies (e.g., CAR-T with checkpoint inhibitors or oncolytic viruses), and the refinement of preclinical models to better predict clinical outcomes. The conditional approval of G47Δ virus in Japan, noted by Yasinjan et al., signals growing regulatory momentum for novel immunotherapeutics in CNS tumors.

## 3 Conclusion

This Research Topic brings together diverse yet interconnected strategies aimed at improving the effectiveness of immunotherapies in pediatric brain cancer. From molecular profiling and preclinical modeling to CAR-T engineering and neoantigen targeting, the contributions reflect a maturing field that is increasingly data-driven, mechanistically informed, and clinically ambitious.

The collective insights emphasize the importance of tailoring immunotherapy to the unique biology of pediatric brain tumors, leveraging subgroup-specific vulnerabilities, and overcoming the structural and immunological barriers of the CNS. Continued innovation in delivery methods, cell engineering, and model systems will be essential to translate these advances into durable clinical benefit.

We call on the research community to prioritize pediatric-specific approaches, foster interdisciplinary collaboration, and support clinical trials that reflect the complexity and diversity of these diseases. Only through such concerted efforts can we move closer to realizing the full potential of immunotherapy for children with brain cancer.

